# GacA uses two distinct regulatory mechanisms to control the biosynthesis of 2,4-diacetylphloroglucinol in *Pseudomonas protegens* Pf-5

**DOI:** 10.1128/aem.01546-25

**Published:** 2025-11-18

**Authors:** Maria Purnamasari, Marjana Ritu, Xiaogang Wu, Brian Tripet, Qing Yan

**Affiliations:** 1 Department of Plant Sciences and Plant Pathology, Montana State University 169020, Bozeman, Montana, USA; 2 Guangxi Key Laboratory of Agro-Environment and Agro-Product Safety, College of Agriculture, Guangxi University 12664, Nanning, China; 3 Department of Chemistry and Biochemistry, Montana State University 428076, Bozeman, Montana, USA; Centers for Disease Control and Prevention, Atlanta, Georgia, USA

**Keywords:** 2,4-diacetylphloroglucinol, *Pseudomonas*, Gac-Rsm, pea *Aphanomyces *root rot

## Abstract

Antibiotic 2,4-diacetylphloroglucinol (DAPG) is produced by many plant-associated beneficial bacteria of the *Pseudomonas* genus and plays an important role in plant disease control due to its broad antimicrobial activity against different pathogens. DAPG biosynthesis is activated by the conserved GacA/GacS two-component regulatory system. Here, we report that a Δ*gacA* mutant of *Pseudomonas protegens* Pf-5, lacking both DAPG production and pathogen inhibition in culture, controlled pea *Aphanomyces* root rot in a DAPG-dependent manner in the greenhouse. DAPG production of the Δ*gacA* mutant could be restored by exogenous phloroglucinol (PG), the first intermediate in DAPG biosynthesis, by culturing the Δ*gacA* mutant with either PG or PG-producing bacteria. We identified a new Gac-dependent promoter of *phlD*. Gene expression assays demonstrated that GacA is required to activate the *phlD* promoter. *In vitro* binding assays showed that the RNA-binding protein RsmE bound directly to the leader mRNA of *phlA*, another DAPG biosynthetic gene converting PG into DAPG, indicating that GacA regulates *phlA* expression post-transcriptionally. No detectable binding activity was observed between RsmE and the *phlD* leader mRNA. These results show that GacA regulates DAPG biosynthesis at multiple steps via different mechanisms and elucidate a novel layer of Gac-Rsm regulation in secondary metabolism. Targeted PG supplementation and/or partner microbe interactions may help to enhance the disease control efficacy and stability of the DAPG-producing bacteria.

**IMPORTANCE:**

Antibiotic production is important for many beneficial bacteria to inhibit plant pathogens and control plant diseases. Understanding the molecular mechanism of how bacteria regulate antibiotic production can help improve the disease control effect. Previous studies have shown that the production of 2,4-diacetylphloroglucinol (DAPG) is activated by a global regulator GacA in strains of *Pseudomonas* spp. In this work, we found that two different regulatory mechanisms are used by GacA to regulate the DAPG production in *Pseudomonas protegens* Pf-5. Specifically, GacA regulates the expression of *phlD* and *phlA*, two DAPG core biosynthetic genes, at transcriptional and post-transcriptional levels, respectively. DAPG production of the Pf-5 mutant lacking GacA could be restored by amendment of phloroglucinol (PG), an intermediate of DAPG biosynthesis. The Δ*gacA* mutant protected pea plants in soil from root rot disease caused by an oomycete pathogen, *Aphanomyces euteiches,* that can be inhibited by DAPG. Results of this study advanced our understanding of the molecular mechanisms that regulate antibiotic production of plant beneficial bacteria and suggest that PG amendment may be used to improve the disease control stability and efficacy of DAPG-producing bacteria in the field.

## INTRODUCTION

Antibiotic production is essential for many plant-associated beneficial bacteria to inhibit pathogens and control plant diseases (1, 2). Understanding the molecular mechanisms by which bacteria regulate antibiotic production can help us improve the efficacy and stability of the beneficial bacteria in plant disease control.

**Fig 1:**
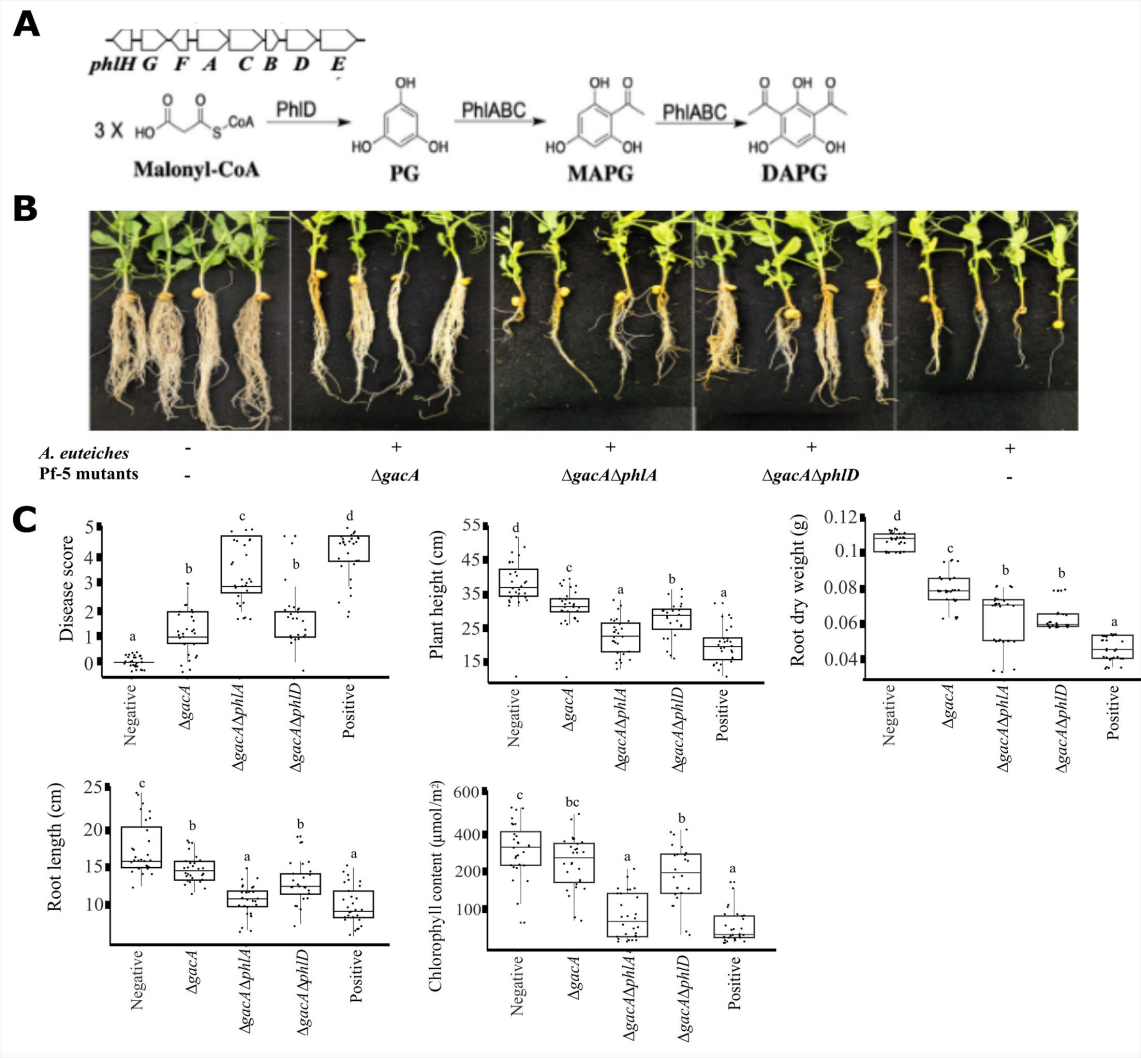
Biocontrol effect of *P. protegens* Pf-5 mutants against pea *Aphanomyces* root rot under greenhouse conditions. (**A**) Schematic representation of the DAPG biosynthetic genes and pathway. Malonyl-CoA serves as the starting substrate, and PhlD catalyzes the production of PG, which is subsequently converted into MAPG and then DAPG by the PhlABC enzyme complex. (**B**) Root rot symptoms of pea plants that were inoculated with (+) and without (−) the pathogen *Aphanomyces euteiches* and Pf-5 mutants. Each treatment had seven pots with four seeds per pot, and the experiment was repeated four times independently. Photos of representative plants are shown. (**C**) Disease severity and other plant growth parameters, including plant height, root weight and length, and leaf chlorophyll content. Positive and negative controls indicate the pea plants inoculated with and without the pathogen, respectively. Data are presented as mean ± SE, with different letters indicating statistically significant differences between treatments (ANOVA, Tukey’s *post hoc* test, *P* < 0.05).

DAPG (syn. 2,4-diacetylphloroglucinol) is a phenolic antibiotic produced by different plant-associated *Pseudomonas* species of worldwide origin and is of interest in plant disease control because of its toxicity to a variety of plant pathogens, including bacteria, fungi, and oomycetes (3–5). Biosynthesis of DAPG requires four core biosynthetic genes, *phlACBD,* that are clustered in the genomes (Fig. 1A) (6, 7). PhlD is a type III polyketide synthase that initiates the DAPG biosynthesis and produces phloroglucinol (PG) from substrate malonyl-CoA (8, 9). PG is then acetylated to monoacetylphloroglucinol (MAPG) and DAPG by the acetyltransferase complex PhlABC (10, 11).

DAPG production of *Pseudomonas* spp. is strongly regulated by the Gac-Rsm signal cascade, which consists of the GacS/GacA two-component regulatory system, the small regulatory RNAs, and several RNA-binding proteins. Gac-Rsm regulates the expression of target genes mainly at post-transcriptional levels (12). The RNA-binding proteins RsmA and RsmE bind to leader mRNAs of target genes and repress their translation (13–15). The response regulator GacA, activated by the sensor kinase GacS, induces the expression of small regulatory RNAs (*rsmX*, *rsmY*, and *rsmZ*), which sequester RsmA/E, thereby relieving the translational repression (16–18). Production of DAPG is positively regulated by GacS/GacA and the small regulatory RNAs but repressed by RsmA/E (18–20). Nucleotide substitutions in the predicted RsmA/E-binding sites upstream of *phlA* reduced the repressive effect of RsmA/E on *phlA* translation, suggesting that *phlA* is likely a direct target of the Gac-Rsm system (18, 21). However, the direct evidence that RsmA/E binds to the *phlA* leader mRNA is still missing. Moreover, it remains unknown if a similar mechanism is used by Gac-Rsm to regulate the expression of the other core biosynthetic genes, *phlBCD*. The mechanism regulating the expression of *phlD* is practically interesting because the *phlD* gene plays an important role in the DAPG biosynthesis: PhlD initiates DAPG biosynthesis by converting the substrate malonyl-CoA into the first intermediate PG (Fig. 1A). Additionally, PG has a broad impact on bacteria by regulating hundreds of genes’ expression even beyond the DAPG biosynthesis (22, 23).

*Pseudomonas protegens* Pf-5 is a soil bacterium that can protect plants from various bacterial and fungal diseases (24–28). Pf-5 produces at least eight antibiotics, including DAPG, pyoluteorin, pyrrolnitrin, hydrogen cyanide, orfamide A, rhizoxin, toxoflavin, and protegenin (29–33). We previously reported that DAPG inhibited the growth of the oomycete pathogen *Aphanomyces euteiches* and was required for Pf-5 to control pea *Aphanomyces* root rot disease under greenhouse conditions (34). Mutation of *gacA* in Pf-5 resulted in the loss of inhibition of *A. euteiches* in culture (34), which is expected given the important role of GacA in the expression of antibiotics, including DAPG (19, 35). Surprisingly, in this study, we found that the Δ*gacA* mutant of Pf-5 controlled the pea *Aphanomyces* root rot disease in a DAPG-dependent manner. Further investigations revealed that Gac-Rsm regulates the expression of *phlD* via a mechanism different from its regulation of *phlA* in Pf-5. The potential implications of our findings in improving disease control efficacy and stability of beneficial bacteria were discussed.

## RESULTS

### Δ*gacA* mutant mitigated the damages of pea *Aphanomyces* root rot

The disease control effect of the Pf-5 Δ*gacA* mutant on pea *Aphanomyces* root rot was evaluated in greenhouse pot assays. Pea seedlings treated with the Δ*gacA* mutant showed less root rot symptoms than the plants treated only with the pathogen (Fig. 1B). The result of the disease assessment indicated that the Δ*gacA* mutant significantly reduced the disease severity compared to the pathogen control (Fig. 1C). Moreover, pea seedlings treated with the Δ*gacA* mutant had a significant increase in plant height, leaf chlorophyll content, root length, and root dry weight than the plants treated with the pathogen only (Fig. 1C). These results showed that the Δ*gacA* mutant could protect pea seedlings from the damage caused by *A. euteiches* in greenhouse pot assays. The results were unexpected because GacA is known to be essential to activate the production of antibiotics, including DAPG in Pf-5 (19, 35), and DAPG is required for Pf-5 to inhibit *A. euteiches* in culture and control the disease in soil (34).

### DAPG is needed in the biocontrol activity of Δ*gacA* mutant

To test if DAPG production is required for the Δ*gacA* mutant to control the disease, a mutation of *phlA*, which encodes an acyl carrier protein synthase for DAPG biosynthesis (Fig. 1A), was introduced into the Δ*gacA* mutant. The generated Pf-5 derivative strain, called Δ*gacA*Δ*phlA* mutant, had a significantly reduced biocontrol effect compared to the Δ*gacA* mutant (Fig. 1B and C). These results suggest that DAPG is required for the Δ*gacA* mutant to control *A. euteiches* in the greenhouse pot assays.

### PG restored DAPG production in the Δ*gacA* mutant

**Fig 2:**
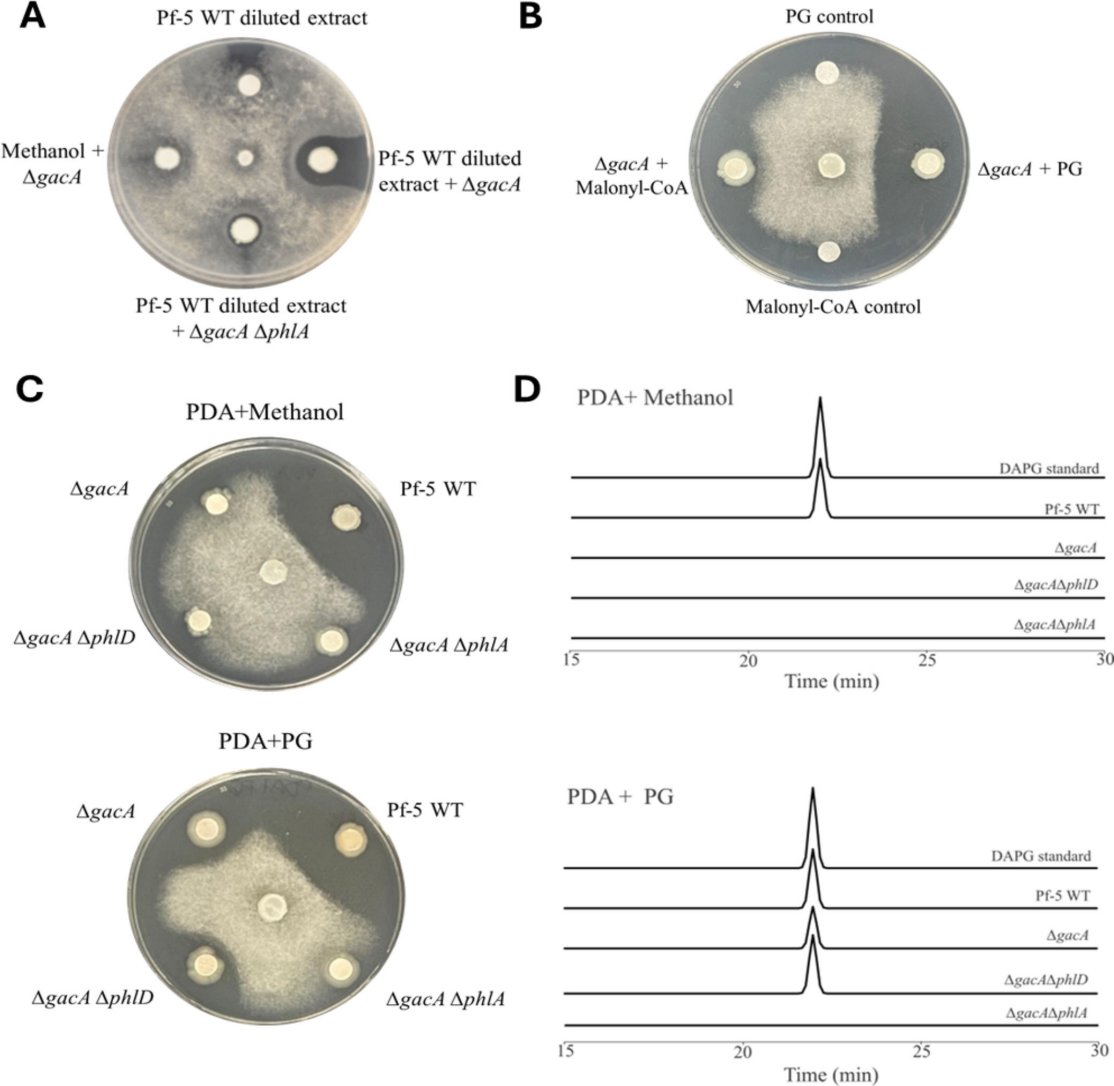
PG restored DAPG production in the ∆*gacA* mutant and inhibition against *A. euteiches*. (**A**) and (**B**) A plug of *A. euteiches* was inoculated at the center of the PDA plate. Pf-5 derivatives, including the Δ*gacA* mutant and the Δ*gacA*Δ*phlA* mutant, were inoculated on filter paper discs that were amended with diluted culture extracts of the wild-type Pf-5 (**A**), malonyl-CoA (100 µg), or PG (100 µg) (**B**). The impact of these compounds and the diluted Pf-5 culture extracts on pathogen growth was assessed. Methanol was included as a solvent control. (**C**) and (**D**) The inhibition against *A. euteiches* (**C**) and the DAPG production analyzed by high-performance liquid chromatography (HPLC) (**D**) of Pf-5 and its derivatives on PDA plates amended with either methanol control (top panel) or PG (bottom panel) (50 µg/mL). Pf-5 WT: wild-type Pf-5 strain. The growth inhibition assays were repeated at least three times. The HPLC assay of DAPG production was repeated two times.

The result that the Δ*gacA* mutant controlled pea root rot in a DAPG-dependent manner implies an unknown mechanism used by Gac-Rsm to regulate DAPG biosynthesis. We hypothesized that the Δ*gacA* mutant has a partially compromised DAPG biosynthesis pathway that can be restored by compounds related to DAPG biosynthesis. To test this hypothesis, the Δ*gacA* mutant was treated with culture extracts of the wild-type strain Pf-5 at a dilution causing no obvious toxic effect to the growth of *A. euteiches* on culture plates (Fig. 2A). *A. euteiches* was inhibited by the Δ*gacA* mutant, which was grown on a filter paper amended with culture extracts of wild-type Pf-5 (Fig. 2A), suggesting that the production of antibiotics by the Δ*gacA* mutant was restored by non-toxic metabolites of Pf-5. Compared to the Δ*gacA* mutant, the Δ*gacA*Δ*phlA* mutant showed reduced inhibition of *A. euteiches* even when amended with Pf-5’s culture extracts, suggesting that DAPG contributed to the inhibition, which is consistent with the report that DAPG is toxic to *A. euteiches* (34). These results support our hypothesis and suggest that the partially compromised DAPG biosynthesis of the Δ*gacA* mutant could be restored by a non-toxic compound that is produced by the wild-type Pf-5.

To identify the metabolites produced by Pf-5 that restored DAPG production of the Δ*gacA* mutant, we tested malonyl-CoA and PG, the substrate and the first intermediate, respectively, for DAPG biosynthesis (Fig. 1A), in the inhibition assay against *A. euteiches*. Neither of the two compounds was toxic to *A. euteiches* (Fig. 2B). Amendment of PG, but not malonyl-CoA, restored the Δ*gacA* mutant’s ability to inhibit *A. euteiches*. To confirm PG’s effect on the inhibition activity of the Δ*gacA* mutant, PG was added directly to the culture medium in the pathogen inhibition assay. As shown in Fig. 2C, the Δ*gacA* mutant inhibited *A. euteiches* on the culture plates added with PG but not on the plates without PG. No obvious inhibition of *A. euteiches* by the Δ*gacA*Δ*phlA* mutant was observed on culture plates with or without PG. The production of DAPG was validated by high-performance liquid chromatography (HPLC) analysis. As expected, wild-type Pf-5 produced DAPG on culture plates. However, DAPG was detected in culture extracts of the Δ*gacA* mutant grown on the plates added with PG but not on the plates added with the solvent methanol (Fig. 2D).

### GacA positively regulates the expression of PhlD

The result that PG, but not malonyl-CoA, restored inhibition of *A. euteiches* and DAPG production in the Δ*gacA* mutant suggests that the Δ*gacA* mutant likely lacks the activity of converting malonyl-CoA into PG, which is catalyzed by PhlD (Fig. 1A) (8). We thus hypothesized that GacA activates the expression of *phlD*. To test this hypothesis, we made a Δ*gacA*Δ*phlD* mutant and assessed its biocontrol effect on pea root rot, as well as its inhibition activity against *A. euteiches* with and without PG amendment. We predicted that the Δ*gacA* mutant would behave similarly to the Δ*gacA*Δ*phlD* mutant if GacA activates the expression of *phlD*. Results showed that the Δ*gacA*Δ*phlD* mutant and the Δ*gacA* mutant had a similar activity in disease control (Fig. 1B and C), pathogen inhibition (Fig. 2C), and DAPG production (Fig. 2D), suggesting that mutation of *gacA* led to a lack of PhlD expression.

**Fig 3:**
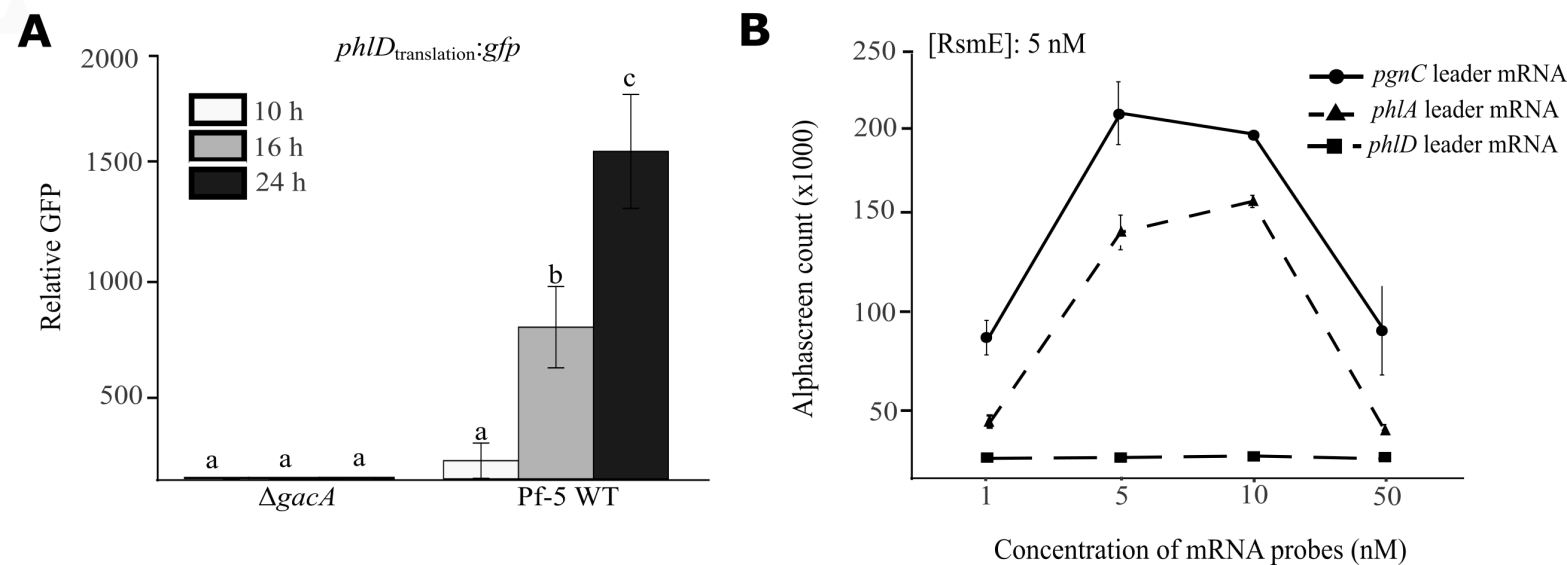
GacA positively regulates PhlD expression through an RsmE-independent mechanism. (**A**) The wild-type Pf-5 and the Δ*gacA* mutant that contain the translational reporter construct p*phlD*_translation_:*gfp* were cultured in PDB medium for 24 h. GFP activity of the reporter strains was measured at 10, 16, and 24 h after inoculation and normalized by bacterial cell density (OD_600_). Bars represent mean ± SE from three independent biological replicates. Pf-5 WT: wild-type Pf-5 strain. Statistical analysis was performed using a two-tailed Student’s *t*-test. (**B**) Interactions between the RsmE protein and the leader mRNA of *phlA* or *phlD* were assessed by AlphaScreen assay. Fifteen microliters of the mRNA probes with different concentrations (1–50 nM) was mixed with the RsmE protein solution (5 nM). The AlphaScreen counts were recorded. The known binding of RsmE to the *pgnC* leader mRNA was included as a positive control. Data are mean ± SD of three biological replicates.

Given that Gac-Rsm regulates the expression of target genes mainly at post-transcriptional levels, mutation of *gacA* may decrease the translation of *phlD*. To evaluate the expression of *phlD*, we made a translational reporter construct of *phlD* by fusing the first six codons of *phlD* in-frame with a *gfp* gene lacking the start codon. The generated reporter construct p*phlD*_translation_:*gfp* was transferred into the wild-type Pf-5 and the Δ*gacA* mutant, and green fluorescence protein (GFP) activity was measured. Compared to the wild-type Pf-5, the Δ*gacA* mutant had a significantly lower GFP activity (Fig. 3A), supporting the hypothesis and indicating that GacA positively regulates the translation of *phlD*.

GacA is known to regulate the expression of target genes by inducing the expression of small RNAs that sequester the RNA-binding proteins, which relieves the translational repression of the target genes exerted by the RNA-binding proteins (12). To investigate if a similar mechanism is used by GacA to regulate the expression of PhlD, the binding activity of purified RNA-binding protein RsmE to the leader mRNA of *phlD* (*phlD*_LmRNA_) was measured. No detectable binding activity was observed between RsmE and *phlD*_LmRNA_ (Fig. 3B). As a positive control, a strong binding activity was observed between RsmE and the leader mRNA of *pgnC* (Fig. 3B), which is a known target gene regulated by GacA in Pf-5 (15). Strong binding activity was also observed between RsmE and the leader mRNA of *phlA* (Fig. 3B), which is consistent with the previous reports that the predicted RsmA/E-binding sites upstream of *phlA* play a role in *phlA* translation (18, 21). These results showed that *phlA*, but likely not *phlD*, is a direct target gene of RsmE and suggest that GacA activates the expression of PhlD through an unknown mechanism.

### GacA is required to activate the promoter activity of *phlD*

**Fig 4:**
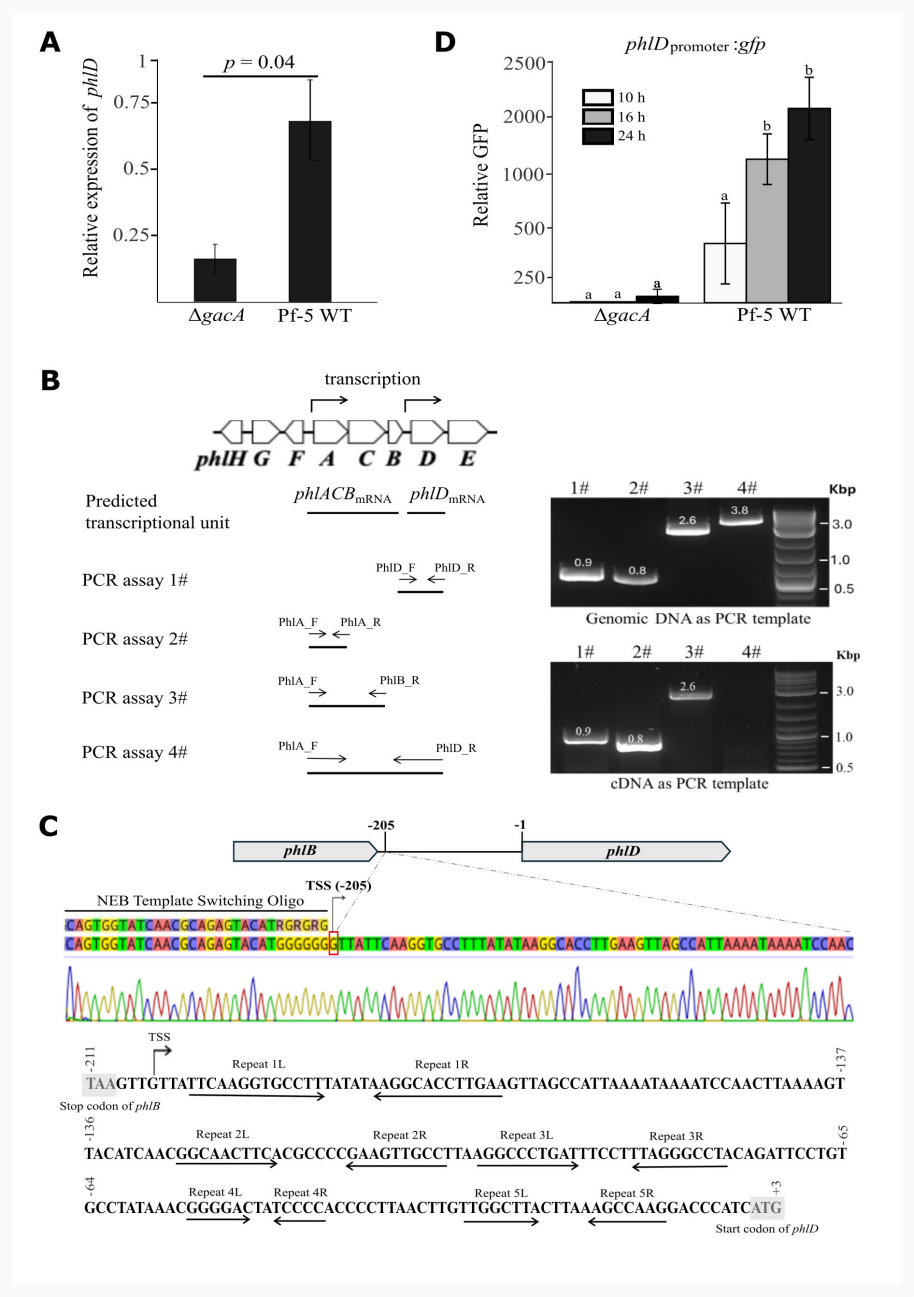
Independent transcription of *phlD* and its regulation by GacA in *P. protegens* Pf-5. (**A**) RT-qPCR analysis of *phlD* expression in wild-type (WT) Pf-5 and ∆*gacA* mutant*.* Total RNA was extracted from Pf-5 WT and Δ*gacA* mutant cultures grown in PDB to the mid-log phase, and the transcription of *phlD* was measured by RT-qPCR analysis. (**B**) Evaluation of the putative transcriptional units of the *phlACBD* gene cluster by RT-PCR. Four PCR assays were conducted using genomic DNA (top gel photo) or cDNA (bottom gel photo) from the wild-type Pf-5 using primer pairs PhlD_F/PhlD_R (assay 1#), PhlA_F/PhlA_R (assay 2#), PhlA_F/PhlB_R (assay 3#), and PhlA_F/PhlD_R (assay 4#). PCR products were separated on a 1.5% agarose gel. (**C**) Mapping the *phlD* transcription start site (TSS) by 5′ rapid amplification of cDNA end analysis. Template-switch cDNA libraries captured a dominant 5′ end at −205 nt upstream of *phlD* (arrow with a vertical line). A representative Sanger sequencing trace of the template switching oligo (TSO)-tagged cDNA is shown, and below it is the DNA sequence of the *phlB-phlD* intergenic region with the TSS and repeat motifs (1–5) indicated by arrows. (**D**) *phlD*_promoter_:*gfp* reporter assay in Pf-5 WT and Δ*gacA*. The reporter strains were cultured in PDB, GFP fluorescence and OD_600_ were measured at 10, 16, and 24 h. The promoter activity was expressed as relative GFP (GFP/OD₆₀₀). Different letters indicate significant differences. Data in (**A**) and (**D**) are mean ± SE of three biological replicates. Statistical analysis was performed with a two-sided Welch’s *t*-test (unpaired).

To investigate the mechanism of how GacA regulates the expression of PhlD, we measured the transcription of *phlD* via real-time quantitative PCR (RT-qPCR). Results showed that the transcription level of *phlD* was significantly lower in the Δ*gacA* mutant than the wild-type Pf-5 (Fig. 4A), suggesting that GacA positively regulates the transcription of *phlD*.

The *phlACBD* gene cluster was reported to be transcribed as a single operon in the DAPG-producing *Pseudomonas fluorescens* strains Q2-87 and F113 (6, 7). To test if *phlD* is transcribed independently in Pf-5, RNAs were extracted from the wild-type strain Pf-5, and the presence of a single *phlACBD* transcript or separate *phlACB* and *phlD* transcripts was assessed via reverse transcription followed by four PCR assays (Fig. 4B). Results showed that the *phlACB* and *phlD* transcripts, but not the *phlACBD* transcript, were detected from the extracted RNAs (Fig. 4B), thus supporting that *phlD* is independently transcribed in Pf-5. Moreover, a putative transcriptional start site was found to be 205 nucleotides upstream from the start codon of *phlD* through 5′ rapid amplification of cDNA end (RACE) analysis (Fig. 4C).

To validate the *phlD* promoter, a DNA fragment containing the intergenic region between *phlB* and *phlD* was amplified by PCR and fused to a promoterless *gfp*. The generated reporter construct p*phlD*_promoter_:*gfp* was transferred into the wild-type Pf-5 and the Δ*gacA* mutant. Strong GFP activity was observed in the wild-type Pf-5 containing the p*phlD*_promoter_:*gfp* construct (Fig. 4D), confirming the *phlD* promoter activity. Compared to the wild-type Pf-5, the Δ*gacA* mutant had a significantly lower GFP activity, suggesting that GacA positively regulates the promoter activity of *phlD*. Overall, these results show that *phlD* has its own promoter, which is positively regulated by GacA in Pf-5.

### Bacterial interaction restored DAPG production in the Δ*gacA* mutant

**Fig 5:**
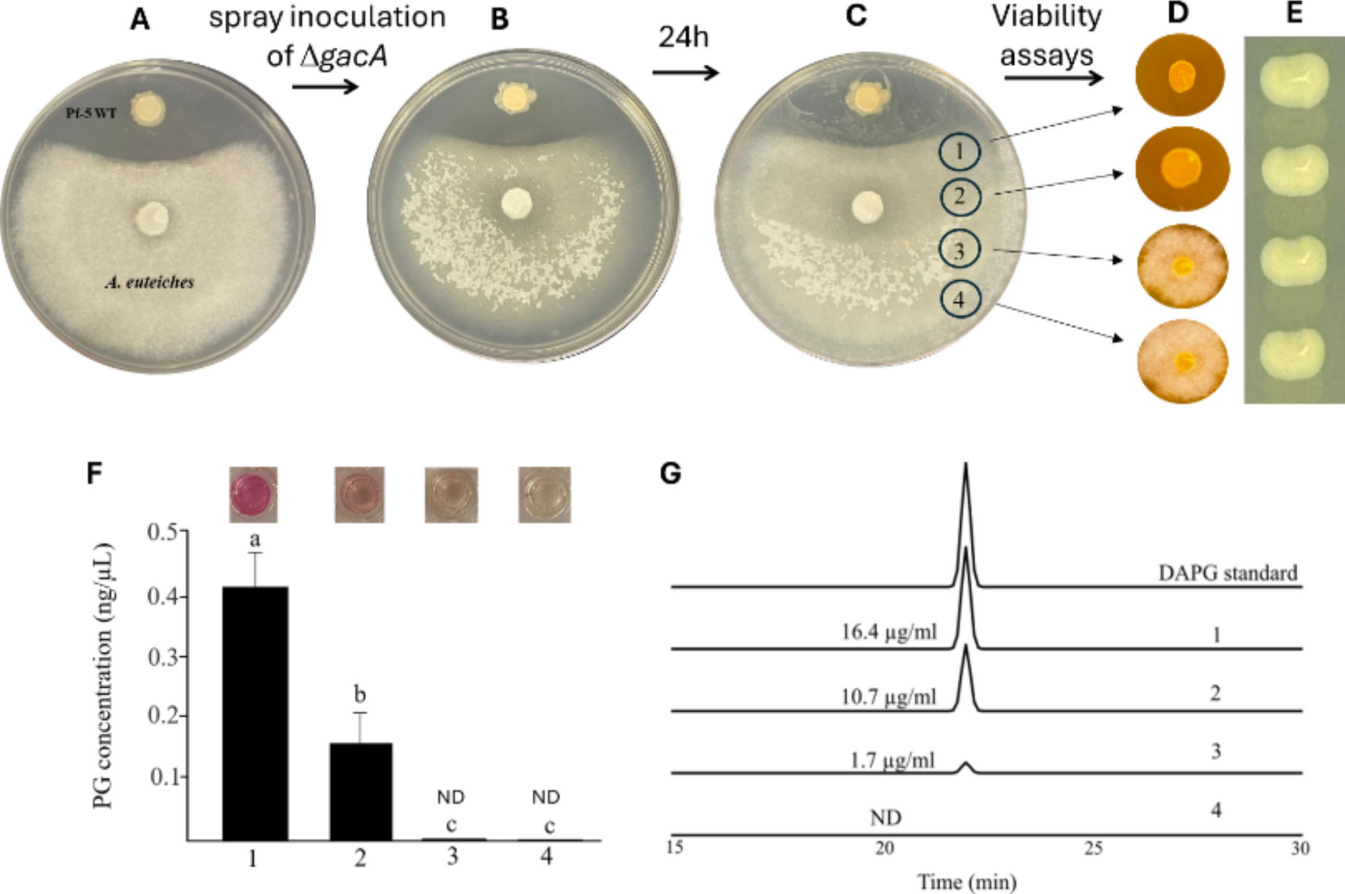
Bacterial interaction restored DAPG production in the Δ*gacA* mutant of *P. protegens* Pf-5. (**A**) Co-culture of Pf-5 wild-type (WT) and *A. euteiches* on PDA plates. A plug of *A. euteiches* was placed at the center, and Pf-5 WT was inoculated at a distance of 2.5 cm. (**B**) After 72 h, a cell suspension of Δ*gacA* (OD_600_ = 1) was sprayed across the entire hyphal area. (**C**) Plates were incubated for another 24 h; four hyphal regions, including regions 1 and 2 (wet morphology) close to the Pf-5 WT colony and regions 3 and 4 (normal morphology), were sampled to test the viability of the hyphae and the bacterium. (**D**) Hyphal viability assay: agar plugs from regions 1–4 were transferred to fresh PDA and monitored for regrowth. (**E**) The presence of Δ*gacA* in the hyphae was tested by streaking the microbial cultures from each sampled region onto a KB plate. (**F**) PG concentration in extracts from regions 1–4 was quantified colorimetrically; bars are mean ± SE (*n* = 3); different letters denote significant differences (one-way ANOVA, Tukey’s HSD, *p* < 0.05). “ND” indicates not detectable. Insets show photos of representative samples. (**G**) HPLC analysis of DAPG in culture extracts from regions 1–4 and a DAPG standard, traces correspond to regions 1–4 (top to bottom). All assays were repeated at least twice.

We hypothesized that the Δ*gacA* mutant can produce DAPG in interactions with PG-producing organisms, such as bacteria, via metabolite cross-feeding. To test this hypothesis, wild-type Pf-5 was inoculated at a distance of approximately 2.5 cm from a fresh plug of *A. euteiches* placed at the center of PDA plates. A clear inhibition zone was observed after 72 h of incubation (Fig. 5A), which is known to be mainly contributed by DAPG produced by Pf-5 (Fig. 2) (34). The Δ*gacA* mutant was then inoculated by spraying its cell suspension into the entire growth area of *A. euteiches* hyphae (Fig. 5B). After an additional 24 h of incubation, hyphae close to the Pf-5 colony collapsed and formed a wet zone that is visibly different from the hyphae growing distantly from the bacterial colony (Fig. 5C). We then assessed hyphal viability by re-inoculating the hyphal plugs on a fresh culture plate. Results showed that the hyphae with wet morphology (hyphal plugs 1 and 2) failed to regrow, but the hyphae with normal morphology (hyphal plugs 3 and 4) regrew well on the new plate (Fig. 5D), indicating that the hyphae near the bacterial colony were dead. The Δ*gacA* mutant was detected in all four culture zones (Fig. 5E). The amounts of PG and DAPG were measured in the four culture zones. Both PG and DAPG were detected in zones close to the Pf-5 colony, and their amounts decreased to undetectable levels in zones distant from the bacterial colony (Fig. 5F and G). Overall, these results support the hypothesis that microbial interactions, potentially via cross-feeding of metabolites, can restore DAPG production in the Δ*gacA* mutant.

## DISCUSSION

The Gac-Rsm is an important regulatory system in the antibiotic production of many plant beneficial *Pseudomonas* species and is well-known to regulate target gene expression mainly post-transcriptionally. Here, we report that Gac-Rsm of *P. protegens* Pf-5 uses different mechanisms to regulate the expression of the DAPG core biosynthetic genes *phlA* and *phlD*: the regulation of *phlA* is directly through the RNA-binding protein RsmE at post-transcriptional level; the regulation of *phlD* is likely indirect via the *phlD* promoter.

A previous study showed that a single transcriptional unit *phlACBD* was detected in the bacterial RNAs via Northern blot analysis in *P. fluorescens* Q2-87 (6), suggesting that the *phlA* promoter drives the expression of *phlACBD* in Q2-87. The *phlA* promoter, since then, has been identified in different *Pseudomonas* species and was used to estimate the level of DAPG biosynthesis (7, 34, 36, 37). In this study, we found that Pf-5 carries an independent *phlD* promoter, which is supported by several lines of evidence, including the detection of *phlD* transcriptional unit via RT-qPCR analysis, the identification of a putative transcription start site of *phlD* via 5′ RACE analysis, and the direct observation of *phlD* promoter activity in GFP-based reporter assays (Fig. 4). DNA sequences of the *phlB-phlD* intergenic region differ markedly between Pf-5 and Q2-87 (data not shown). Phylogenetic analysis, based on the *phlACBD* nucleotide sequences, of 24 different *Pseudomonas* strains revealed two major clades, including one clade of the *Pseudomonas corrugata* group (harboring Q2-87) and a second clade of the *P. protegens* group (harboring Pf-5) (4). Sequence variations of the *phlF-phlA* intergenic region between the *P. protegens* group strain (*P. protegens* CHA0) and the *P. corrugata* group strains (*P*. spp. strains Q2-87 and F113) have been noticed, although the impact of these variations on the expression of DAPG biosynthesis genes was not investigated (7). The variations of regulatory regions between *P. corrugata* and *P. protegens* groups support the previous hypothesis that a diversified regulation pattern for DAPG production may exist among species of *Pseudomonas* (4). Strains of these two groups may differentially regulate DAPG production even in response to the same environmental conditions, such as types of plant, pathogen, and soil. Many DAPG-producing strains of the *P. protegens* group and the *P. corrugata* group were identified to control plant diseases (38–40). Understanding the differences between these two groups in the regulation of *phlACBD*’s expression can help to better use the DAPG producers in disease control. The presence of an independent *phlD* promoter in Pf-5 but not in Q2-87 clearly indicates a diversified regulation pattern for *phlD* expression. It is worth noting that strains of *P. protegens* produce DAPG and another antibiotic, pyoluteorin, which is absent in the DAPG-producing strains of the *P. corrugata* group (38, 41, 42). PG serves as a signal precursor regulating the production of pyoluteorin in a concentration-dependent manner (23, 43). Furthermore, PG influences the expression of hundreds of genes in the genome of Pf-5 (22). Considering the distinct roles of PG in the biosynthesis of DAPG versus pyoluteorin and its broad impact on the bacterial transcriptome, it is not surprising that an independent promoter evolved in *P. protegens* to specifically regulate the expression of *phlD*.

Gac-Rsm is well known to regulate the expression of target genes post-transcriptionally via the RNA-binding protein RsmA and RsmE that bind to motifs around the ribosome-binding site (RBS) of target genes in *P. protegens* (12, 17, 44, 45). No binding activity was detected between the purified RsmE protein and a 120-nt mRNA probe encompassing more than 50 nucleotides upstream and downstream of the putative RBS of *phlD* (Fig. 3B). In comparison, the RsmE protein had a strong binding activity to a 72-nt mRNA probe encompassing around 35 nucleotides upstream and downstream of the putative RBS of *phlA* (Fig. 3B). These results suggest that Gac-Rsm regulates the expression of *phlD* likely in an RsmE-independent manner. We cannot exclude the possibility that RsmA may bind to the *phlD* leader mRNA, although the functional redundancy of RsmA and RsmE has been reported (18, 46, 47). Nevertheless, mutation of *gacA* significantly reduced the *phlD* promoter activity (Fig. 4D), indicating a strong regulation of GacA on the transcription of *phlD*. The mechanism by which GacA activates *phlD* transcription was not investigated. It is possible that GacA controls *phlD* transcription indirectly via regulators controlled by RsmA/E. PhlF is a transcriptional regulator encoded immediately next to *phlACBD* (Fig. 1A). PhlF was found to repress the expression of *phlD* in *P. fluorescens* F113, assessed using a *phlACBD-lacZ* transcriptional reporter (48). The repression of *phlD* expression by PhlF in F113 is likely through the *phlA* promoter, which contains an inverted repeated sequence called the *phO* operator bound directly by PhlF protein (7). A similar *phO* site was found in the intergenic region of *phlF-phlA* but not *phlB-phlD* in Pf-5 (data not shown), implying that PhlF is unlikely to be a direct regulator of *phlD* in Pf-5, although experimental evidence is needed. Despite the fact that Gac-Rsm regulates the expression of target genes mainly post-transcriptionally, GacA can bind directly to promoters and regulate the transcription of target genes. For example, a GacA homolog regulator TzpA regulates the transcription of *vfmE*, a quorum-sensing system regulator, by directly binding to the Gac-box motif in the promoter region of *vfmE* in *Dickeya oryzae* (49). The conserved GacA-binding site was absent in the *phlB-phlD* intergenic region, suggesting that GacA may not directly regulate the *phlD* promoter activity in Pf-5. Overall, the transcriptional regulators that directly control the promoter activity of *phlD* remain undiscovered. Interestingly, inverted repeat sequences were detected in the *phlB-phlD* intergenic region (Fig. 4C), requiring future research to identify the regulators that interact with these motifs and investigate their roles in the GacA-mediated regulation of *phlD* expression.

Spontaneous mutants of plant beneficial *Pseudomonas* lacking GacS and/or GacA were routinely detected in bacterial populations in culture, soil, and during plant-bacteria interactions (50–53). Our result that the Δ*gacA* mutant could regain disease-suppressive activity in soil highlights the complexity of the soil system in influencing plant beneficial traits (such as antibiotic production in this report) and reinforces the importance of evaluating the plant beneficial traits in soil conditions (54, 55). Negative impacts of soil systems on the efficacy and stability of plant beneficial bacteria have been widely acknowledged (56–58). For example, interspecies interactions decreased the production of DAPG in *P. protegens* DTU9.1 (59). Our report suggests that a positive impact was conferred by the soil system, which converted the Δ*gacA* mutant from DAPG nonproducer to DAPG producer. An external source of PG is likely needed by the Δ*gacA* mutant to restore DAPG production in soil. This is supported by several results, including (i) mutation of *phlA*, but not *phlD* (responsible for PG production), reduced the disease control effect of the Δ*gacA* mutant in soil (Fig. 1); (ii) PG restored DAPG production and inhibition against *A. euteiches* by the Δ*gacA* mutant in culture (Fig. 2); (iii) interaction of the Δ*gacA* mutant with a PG-producing bacterium killed hyphae of *A. euteiches* (Fig. 5). The sources of PG in the soil system used in this study were not identified but can be microorganisms, root exudates, and/or chemical substrates in the soil (60–62). Considering the accumulation of *gac* mutants in the rhizosphere (53, 63) and the wide availability of PG in nature, it is very likely that the spontaneous *gac* mutants could restore DAPG production in certain natural circumstances. Restoring production of a specific antibiotic (DAPG in this case) of the *gac* mutants may provide benefits to the bacterial population because of the fast growth rate of the *gac* mutants (15, 52) and the competitive advantages conferred by antibiotic production (64, 65). We may boost this natural phenomenon by adding biotic and/or abiotic sources of PG in the rhizosphere to improve disease control efficacy and stability of the DAPG-producing *Pseudomonas*. Overall, these results suggest that the Δ*gacA* mutant can restore DAPG production by acquiring PG from soil environments, which indicates that metabolic cross-feedings can play an important role in influencing the antibiotic production of bacteria during interactions with other microorganisms and/or plant hosts in soils.

The result that RsmE protein could bind the *phlA* leader mRNA *in vitro* (Fig. 3B) suggests that RsmE represses PhlA expression post-transcriptionally in Pf-5, which is in agreement with the previous reports that mutation of predicted RsmA/E-binding sites in the *phlA* upstream region enhanced PhlA expression at post-transcriptional levels in *P*. spp. strains CHA0 and 2P24 (18, 21). According to these results, the Δ*gacA* mutant was expected to lack PhlA expression. Intriguingly, PG restored DAPG biosynthesis of the Δ*gacA* mutant (Fig. 2C), indicating the expression of PhlA in the Δ*gacA* mutant. The necessity of PhlA for the Δ*gacA* mutant to convert PG into DAPG was proved by the result that the Δ*gacA*Δ*phlA* mutant failed to produce DAPG with PG amendment (Fig. 2C). These data support that a GacA-independent mechanism is used by Pf-5 to counteract the RsmE’s repression on PhlA expression, adding another layer of complexity to our current knowledge of the Gac-Rsm regulatory system.

**Fig 6:**
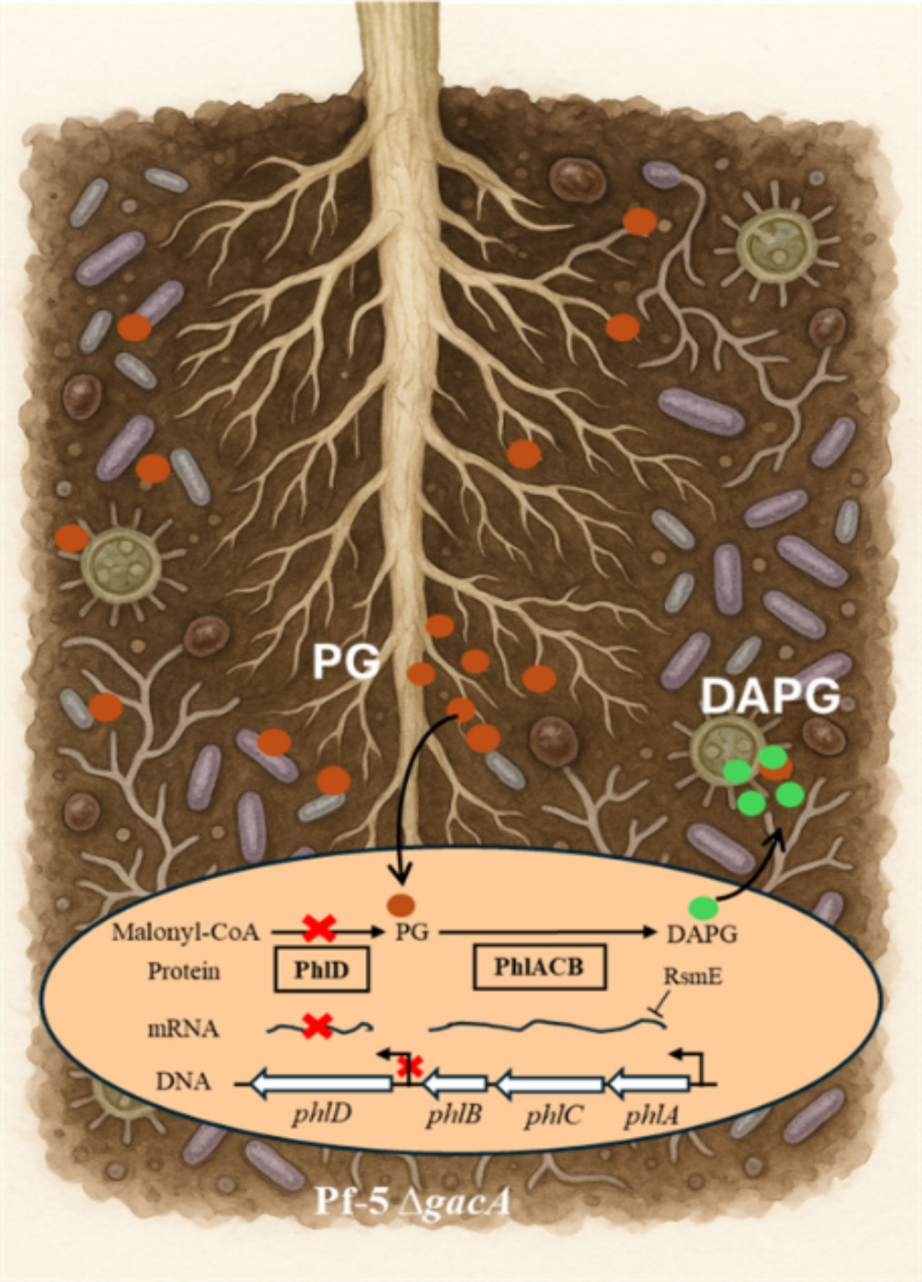
Proposed model of PG-mediated restoration of DAPG production in the Δ*gacA* mutant of Pf-5. The Δ*gacA* mutant cannot produce PG due to the abolished promoter activity of *phlD* that leads to a lack of the PhlD protein. However, co-culturing the Δ*gacA* mutant with exogenous PG (brown sphere), from biotic and/or abiotic resources, restored DAPG (green sphere) production and inhibition to the target pathogen *A. euteiches*, which explains why the Δ*gacA* mutant could control the pea *Aphanomyces* root rot disease in greenhouse pot assays. Although RsmE can bind the leader mRNA of *phlA* and repress its expression at the post-transcriptional level, the repression can be overcome by an unknown mechanism in a GacA-independent manner.

As summarized in Fig. 6, Pf-5 mutants lacking the GacA global regulator could control pea *Aphanomyces* root rot in a DAPG-dependent manner. DAPG production of the Δ*gacA* mutant could be restored by either PG amendments or cross-feeding from PG-producing bacteria. GacA regulates the expression of DAPG core biosynthetic genes through different mechanisms, including directly regulating *phlA* expression post-transcriptionally via RsmE and indirectly regulating *phlD* expression transcriptionally via the *phlD* promoter. Extensive efforts have been made to activate silent biosynthetic gene clusters and to fine-tune active biosynthetic gene clusters for antibiotics and other natural products of microorganisms (66–68). Our finding underscores the genetic plasticity of bacteria and provides a unique example of reactivating a biosynthetic gene cluster in a bacterial mutant that lacks an important global regulator of secondary metabolism.

## MATERIALS AND METHODS

### Microbial strains and cultural conditions

The oomycete and bacterial strains, plasmids, and sequences of oligonucleotides used in this study are listed in Table 1. *Pseudomonas protegens* Pf-5 and its mutants were cultured at 28°C on different media, including potato dextrose agar (PDA, Becton, Dickinson and Company), King’s Medium B (KB) agar, and nutrient broth (NB; Becton, Dickinson and Company) supplemented with 1% glycerol (NBGly) or 2% glucose (NBGlu), with or without 1.5% agar (Difco Agar; Becton, Dickinson and Company, Franklin Lakes, NJ, USA). Liquid cultures were grown with a shaking at 200 rpm. *Aphanomyces euteiches* was cultured at room temperature on PDA plates or Corn Meal Agar (CMA, Sigma Aldrich). Malonyl-CoA and phloroglucinol were purchased from Sigma-Aldrich (St. Louis, MO, USA) and dissolved in methanol before use.

**TABLE 1:** Bacterial strains, plasmids, and primers used in this study

Strains, plasmids, and primers	Genotypes, relevant characteristics, and DNA sequences^*a*^	Reference or source
*Aphanomyces euteiches*	An oomycete pathogen isolated from a pea *Aphanomyces* root rot disease sample in Montana.	Lab stock
BL21(DE3)	A genetically modified *Escherichia coli* commonly used for protein overexpression, *ompT hsdS*_B_ (rB^-^ mB^-^) *gal dcm*	NEB
*P. protegens* strains		
Wild-type Pf-5	Pf-5 is a soil bacterium that produces DAPG, pyoluteorin, pyrrolnitrin, hydrogen cyanide, orfamide A, rhizoxin, toxoflavin, and protegenin. Assigned ID is LK099.	(24)
∆*gacA*	A Pf-5 derivative, which has an unmarked in-frame deletion of *gacA*. Assigned ID is JL4975.	(31)
∆*gacA*∆*phlA*	A Pf-5 derivative, which has an unmarked in-frame deletion of *gacA* and *phlA*. Assigned ID is LK546.	This study
∆*gacA*∆*phlD*	A Pf-5 derivative, which has an unmarked in-frame deletion of *gacA* and *phlD*. Assigned ID is LK585.	This study
Plasmids		
pME6010	An expression vector in *Pseudomonas protegens*. To make gene complementation constructs of Pf-5. Tc^r^	(69)
pME6010-*phlA*	pME6010 containing the sequence upstream of the *phlA* start codon. Serve as a template to create *phlA* leader mRNA. Km^r^	This study
pME6010-*phlD*	pME6010 containing the sequence upstream of the *phlD* start codon. Serve as a template to create *phlD* leader mRNA. Km^r^	This study
pME6010-*pgnC*	pME6010 containing the wild-type *pgnC* gene. Serve as a template to create *pgnC* leader mRNA. Km^r^	(15)
pEX18Tc	A suicide vector in *Pseudomonas protegens*. To make deletion and gene replacement constructs of Pf-5. *sacB*^+^ Tc^r^	(70)
pEX18Tc-Δ*phlA*	pEX18Tc containing an in-frame deleted version of the *phlA* gene. To delete *phlA* in the chromosome of Pf-5.	(43)
pEX18Tc-∆*phlD*	pEX18Tc containing an in-frame deleted version of the *phlD* gene. To delete *phlD* in the chromosome of Pf-5.	(43)
pPROBE-NT	An empty vector to make gene expression reporter constructs. Containing a promoterless *gfp*, Km^r^	(71)
p*phlD*_promoter_-*gfp*	pPROBE-NT containing the *phlD* promoter fused with the promoterless *gfp*. To measure the transcriptional expression of the *phlD* gene, Km^r^	This study
p*phlD*_translation_-*gfp*	pPROBE-NT containing the promoter and the first six codons of *phlD* fused in-frame with the promoter-less *gfp*. To measure the translational expression of the *phlD* gene, Km^r^	This study
pMiniT 2.0	An *E. coli* plasmid cloning vector designed for cloning blunt-ended or single-base overhang PCR product using the NEB PCR Cloning Kit. Ap^r^	NEB
**Primers**	Oligonucleotide sequences (5′–3′)	
phlD-TL6_F	CTGCAGGTCGACTCTAGAGGAAATCGTCGATGTGTGCGA	
phlD-TL6_R	AGTGAAAAGTTCTTCTCCTTTACTCATAAGGCAAAGTGTAGACAT	
phlD-PP_F	ATGTCTACACTTTGCCTTATGAGTAAAGGAGAAGAACTTTTCAC	
phlD-PP_R	TCGCACACATCGACGATTTCCTCTAGAGTCGACCTGCAG	
PphlD_F	TATAAGCTTCGGAAATCGTCGATGTGT	
PphlD_R	TATGAATTCAGGAAGTGACGATCACCAT	
KpnI-phlA_F	ATGGTACCAAGAATGGAATCAAGAGGAAAATGAACATGTCTACACTTTGCCTTCC	
KpnI-phlD_F	ATGGTACCTGTGCCTATAAACGGGGAC	
HindIII-phlD_R	TATAAGCTTGGCATTCCAGTGTTCATCG	
PhlA_UTRT7_F	TAATACGACTCACTATAGGGGCATGCCATGGTACCAAGAA	
PhlA_UTRT7_R	CATGACGTGTGGAAGGCAAA	
PhlD_UTRT7_F	TAATACGACTCACTATAGGGCCTGTGCCTATAAACGGGGAC	
PhlD_UTRT7_R	GGTGATCTTGTGGTGGGGA	
PgnC_UTRT7_F	TAATACGACTCACTATAGGGCACAGGGAATAGCGTGGGCAG	
PgnC_UTRT7_R	CGATCCCATGGCACCTTCCAG	
PhlA_R	TGCTTCTGGTACTGCTCGAT	
PhlB_R	GATTTCCGAAGCGATCACCA	
PhlD_F	TTTGCCTTCCACACGTCATG	
PhlD_R	ATCGAACTGCCGCTTGAGTA	
phlD-qF	ACGTACTGATCGTGTCC	
phlD-qR	TGTAGTGCTCGCTCTT	
zwf-qF	TACGAGCGGTTGTTGC	
zwf-qR	ATACCAAGACCTCCCAT	
TSO	GCTAATCATTGCAAGCAGTGGTATCAACGCAGAGTACATrGrGrG	
TSO-F	CATTGCAAGCAGTGGTATCAAC	
*phlD*_gsp	CATCGCCGAACAGCGCCGCG	
*phlD*_gsp2	CAGGAAGTGACGATCACCAT	

^*a*^ Tc, tetracycline (10 µg/mL for *E. coli*, 200 µg/mL for Pf-5); Km, kanamycin (50 µg/mL); and Ap, ampicillin (100 µg/mL).

### Construction of Pf-5 mutants

Pf-5-derived deletion mutants were generated by following our previous method (15). The in-frame deletion constructs pEX18Tc-Δ*phlA* and pEX18Tc-Δ*phlD* were made previously (43). The deletion constructs were confirmed by Sanger sequencing and introduced into the ∆*gacA* mutant via electroporation (1.8 kV, 5 milliseconds) to introduce an in-frame deletion of *phlA* and *phlD* in the genome. The deletion of the *phlA* and *phlD* was confirmed by PCR analysis.

### Preparation of oospores, soil inoculations, and biocontrol assays

To prepare the pathogen inoculum, *A. euteiches* was cultured on CMA plates for 10 days. The mycelial mats were then homogenized in water for 5 minutes, and oospore density was measured using a hemocytometer. The oospore suspension was diluted into greenhouse soil to achieve 100 oospores per gram of soil. An equivalent volume of water was added to the soil as a control. The greenhouse soil used in this study was Sunshine Mix #1 (pH: 5.8, NO_3_-N: 35 ppm, NH_4_-N: 14 ppm, PO_4_-P: 42 ppm, K: 113 ppm; Mg: 90 ppm, Ca: 366 ppm, and Na: 23 ppm), which was aerated and steam pasteurized (70°C, 1 h). The pathogen-inoculated soils were transferred to 10 cm square pots. Field pea seeds (*Pisum sativum L*., cv. Carousel yellow) were sterilized with 3% sodium hypochlorite for 5 minutes, rinsed three times with sterilized distilled water, and air-dried under sterile conditions. To prepare the bacterial inoculum, Pf-5 derivatives were grown on KB plates at 28°C overnight. The bacterial cells were collected from the plates and washed three times with sterile water into a cell suspension with an optical density at 600 nm (OD_600_) of 0.1. Surface-sterilized pea seeds were immersed in the bacterial cell suspension for 3 h at room temperature with gentle agitation before being sown in the pots with prepared soil. Each of the pots was placed in a shallow saucer filled with water to induce disease. Greenhouse conditions were set at 22°C (day) and 18°C (night) with a 16-h photoperiod (6am - 10pm). Each treatment included seven pots with four seeds per pot, and the experiment was performed four times independently. After three weeks of sowing, pea plants were harvested for disease evaluation. Roots were rinsed with tap water to remove soil, and disease severity was scored based on the following symptom descriptive keys: 0, no visible symptoms; 1, minor water-soaking on roots; 2, moderate discoloration (25%–50%) or water-soaking on roots or epicotyls; 3, extensive softening and discoloration (51%–75%) without complete collapse; 4, severe damage (76%–100% root discoloration), including root disintegration; 5, dead plant. The disease index was then calculated to evaluate the disease control effects of the tested Pf-5 strains.

### Assessment of *Aphanomyces euteiches* inhibition

To evaluate the inhibition of Pf-5 derivatives on the growth of *A. euteiches*, a mycelium plug (5 mm in diameter) of a 4-day-old *A. euteiches* culture was placed at the center of the PDA plates. Cells of Pf-5 derivatives were washed by centrifuging to pellet the cells and resuspending the cell pellet in sterile water. The washing process was repeated at least two times. The cells were resuspended in 1 mL of sterile water, and the cell density was adjusted to OD_600_ = 0.1. Three microliters of the bacterial cell suspension was inoculated on the PDA plate approximately 2.5 cm away from the mycelium plug. The plates were incubated at 28°C for 2–3 days before inhibition was recorded by photographing. Each bacterial strain was tested in triplicate, and the experiment was repeated three times.

To test the effect of wild-type Pf-5 culture extracts on the ability of Pf-5 mutants to inhibit *A. euteiches*, Pf-5 wild type was cultured in 50 mL of NBGlu at 28°C with shaking for 24 h. The cultures were extracted with an equal volume of ethyl acetate. The organic phase was collected, evaporated to dryness, and the resulting residues were resuspended in 400 µL of methanol. The extract suspension was diluted two times by mixing 30 µL of the methanol stock with 30 µL of pure methanol. A 5 µL aliquot of the diluted extract was applied onto a sterilized filter paper disc (6 mm in diameter), allowed to air-dry, and then placed onto the PDA plates. Pf-5 mutants were inoculated by adding 3 µL of the bacterial cell suspensions (OD_600_ = 1.0) on the PDA plate approximately 2.5 cm away from the mycelium plug. A similar method was used to test the effect of purified compounds. Specifically, stock solutions of Malonyl-CoA and PG were prepared, respectively, at concentrations of 20 mM in Milli-Q water and methanol. The compound was applied by adding 5 µL of the compound solution to a filter paper disc, drying it, and placing it on the PDA plate. Subsequently, 3 µL of the bacterial suspension was added directly onto the filter paper disc. The inhibition of *A. euteiches* was assessed as described above.

### Quantification of DAPG by HPLC analysis

Quantification of DAPG from bacterial cultures was performed as previously described (23, 25). Ten agar plugs (~7 mm diameter) were collected and transferred into a flask containing 4 mL of acetone:water (1:1, vol/vol). The slurry was shaken at room temperature for 1 h, then the supernatant was collected. The acetone was removed under vacuum in Vacufuge plus (Eppendorf), and the residual aqueous phase was subjected to two rounds of ethyl acetate extraction, followed by vacuum drying. The dried extracts were resuspended in methanol and subsequently analyzed by high-performance liquid chromatography (HPLC) to quantify DAPG production.

The HPLC analysis was performed using an Agilent 1100 system equipped with a quaternary pump, vacuum degasser, autosampler, column oven maintained at 30°C, and a diode array detector. Metabolite separation was carried out using a C18 reverse-phase column (4.6 × 150 mm, 5 µm; Agilent Technologies) at a flow rate of 1 mL/minute. Solvent A consisted of water with 0.1% (vol/vol) formic acid, and solvent B consisted of acetonitrile with 0.1% (vol/vol) formic acid. The column was initially equilibrated in 90% solvent A and 10% solvent B, a condition that was held for 2 minutes following sample injection. A linear gradient was then applied to reach 100% solvent B over 28 minutes, maintained for 6 minutes, followed by a return to 90% A/10% B over 2 minutes. The column was re-equilibrated for an additional 6 minutes before the next run. Under these conditions, DAPG was detected at retention times of approximately 22.7 minutes. Data acquisition and instrument control were performed using ChemStation software (version B.04.03; Agilent, Santa Clara, CA, USA). DAPG was quantified by integrating the peak area at 300 nm and converting the peak areas to concentration using a standard curve generated with purified DAPG.

### Construction of reporter constructs and GFP activity measurement

Two GFP reporter constructs, p*phlD*_translation_:*gfp* and p*phlD*_promoter_:*gfp*, were made in this study to assess the expression of *phlD* at post-transcriptional and transcriptional levels, respectively. To make p*phlD*_translation_:*gfp*, a 355-bp DNA fragment, which contains the promoter and the first six codons of *phlD,* was PCR amplified from the Pf-5 genome using primers phlD-TL6_F and R (Table 1). The vector pPROBE-NT was PCR amplified using primers phlD-PP_F and R, and the PCR product was digested with *Bam*HI. The purified two DNA fragments were assembled using NEBuilder HiFi DNA Assembly Master Mix (NEB, catalog no. E2621L) to make p*phlD*_translation_:*gfp*. To make p*phlD*_promoter_:*gfp*, a 673-bp DNA fragment containing the *phlD* promoter region was amplified from the Pf-5 genome using primers PphlD_F and PphlD_R (Table 1), digested with *Eco*RI and *Hin*dIII, and ligated into pPROBE-NT to generate reporter construct p*phlD*_promoter_:*gfp*. The reporter constructs were confirmed by Sanger sequencing analysis and transformed into wild-type Pf-5 and ∆*gacA* mutant via electroporation (1.8 kV, 5 milliseconds) to make the reporter strains. Pf-5 containing the empty vector pPROBE-NT was used as a control for the reporter strains.

To measure the GFP activity of Pf-5 reporter strains, wild-type Pf-5 and ∆*gacA* containing the above-made construct or the empty vector were cultured overnight on KB plates plus kanamycin at 28°C. The cells were washed in 1 mL of sterile water with an OD_600_ of 1.0 (as described above) and then used to inoculate 200 µL of PDB to achieve a starting OD_600_ of 0.01. Each strain was grown in three wells of a 96-well plate, which was incubated at 28°C with shaking at 180 rpm in a SPARK multimode Microplate Reader (TECAN, Switzerland). Bacterial growth was monitored by measuring the OD_600_. The GFP activity of bacteria was monitored by measuring emission at 535 nm with an excitation at 485 nm and corrected by subtracting fluorescence background emitted by a control strain carrying the empty vector pPROBE-NT. The GFP value was divided by the corresponding OD_600_ to determine the relative GFP level. Each reporter strain was evaluated with at least three replicates. The experiment was repeated two times independently.

### Overexpression and purification of His-tagged RsmE protein

The RsmE protein was overexpressed and purified as previously described (15). The pET28a-RsmE plasmid was transformed into *E. coli* BL21(DE3), and protein expression was induced with 1 mM IPTG at an OD_600_ of 0.6. Cultures were incubated overnight at 37°C. Cells were lysed by sonication in binding buffer (20 mM sodium phosphate and 300 mM NaCl, pH 7.4), and cell debris was removed by centrifugation at 15,000 rpm for 30 minutes at 4°C. The His-tagged RsmE protein was purified using NEB Express Ni Resin (New England Biolabs) as per the manufacturer’s protocol. The protein was eluted in elution buffer (20 mM sodium phosphate, 300 mM NaCl, and 500 mM imidazole, pH 7.4), dialyzed against imidazole-free buffer, and concentrated using Macrosep Advance Centrifugal Devices (3 kDa MWCO). Protein concentration was determined by measuring absorbance at 280 nm using a NanoDrop ND-1000 spectrophotometer.

### Synthesis of the *phlA*, *phlD,* and *pgnC* leader mRNAs

To isolate the DNA corresponding to the *phlA* leader sequence upstream of the start codon, PCR amplification was performed using the forward primer PhlA_UTRT7_F, incorporating the T7 RNA polymerase promoter, and the reverse primer PhlA_UTRT7_R (Table 1). The construct pME6010-*phlA* served as the template, producing a 72-bp PCR product corresponding to the *phlA* leader mRNA, including 42 bp upstream of the first nucleotide of *phlA* and 30 bp downstream. To make pME6010-*phlA*, a 1,534-bp DNA fragment containing the *phlA* leader region was amplified using the primers KpnI-phlA_F and HindIII-phlD_R (Table 1). The resulting fragment was digested with *Kpn*I and *Hind*III, then ligated into a similarly digested pME6010 vector. To obtain the *phlD* leader sequence, primers PhlD_UTRT7_F, which includes a T7 RNA polymerase promoter, and PhlD_UTRT7_R (Table 1) were used with pME6010-*phlD* as a template. This yielded a 120-bp PCR product corresponding to the *phlD* leader mRNA, comprising 69 bp upstream of the first nucleotide of *phlD* and 51 bp downstream. The pME6010-*phlD* was constructed by amplifying a 1,247-bp DNA fragment containing the *phlD* leader region fused to the full-length *phlD* coding sequence using primers KpnI-phlD_F and HindIII-phlD_R (Table 1). After digestion with *Kpn*I and *Hind*III, the fragment was ligated into the digested pME6010 vector to produce pME6010-*phlD*. For *pgnC*, PCR amplification was performed using primers PgnC_UTRT7_F and PgnC_UTRT7_R with the construct pME6010-*pgnC* as the template (15). This resulted in a 156-bp PCR product. The mRNA was synthesized *in vitro* from the PCR products using the HiScribe T7 High Yield RNA Synthesis Kit (New England Biolabs). Each transcription reaction included 1 µg of purified PCR product, RNA NTPs (7.5 mM each of ATP, CTP, and GTP, 5 mM UTP, and 2.5 mM Biotin-16-UTP [Roche, Sigma]), 5 mM DTT, T7 RNA Polymerase Mix, and the supplied reaction buffer. Reactions were incubated overnight at 37°C. The RNA was purified using the Monarch RNA Cleanup Kit (New England Biolabs, T2040L) following the manufacturer’s instructions. The purified RNA was quantified by measuring absorbance at 260 nm, verified as a single band via electrophoresis on a 1.5% agarose gel, and stored at −80°C.

### AlphaScreen assay

The interaction between the leader mRNAs and RsmE was assessed via AlphaScreen assays, adapted from the method described previously (72). The assay was conducted in a buffer containing 30 mM HEPES (pH 7.5), 100 mM KCl, 40 mM NaCl, 10 mM ammonium acetate, 10 mM guanidinium hydrochloride, 2 mM MgCl_2_, 0.5 mM EDTA, 3% DMSO, and 0.01% NP-40, using an AlphaScreen histidine (nickel chelate) detection kit (Perkin Elmer). The reactions were performed in 384-well white polystyrene plates (Corning) with a final volume of 30 µL per well. Each well contained His-tagged RsmE protein and biotinylated *phlA*, *phlD,* or *pgnC* leader mRNA (15 µL), which were incubated in the dark for 30 minutes at room temperature. Next, acceptor beads (10 µg/mL) were added, followed by a 30-minute incubation. Subsequently, donor beads (10 µg/mL) were added, and the mixture was incubated for an additional 30 minutes. The AlphaScreen signal was measured using a Spark Multimode Microplate Reader (Tecan) equipped with the HTS AlphaScreen module. Each reaction was performed in triplicate.

### RNA isolation and purification

Total bacterial RNAs were extracted from Pf-5 cultures using the RNeasy Mini Kit (Qiagen) according to the manufacturer’s protocol. Overnight cultures grown in PDB medium were centrifuged to pellet cells, and the pellets were resuspended in Buffer RLT. RNA was purified following the kit’s instructions. To remove residual DNA, samples were treated with the TURBO DNA-free Kit (Thermo Fisher Scientific) according to the manufacturer’s protocol. The absence of DNA contamination was verified by PCR. The RNA was purified using the Monarch RNA Cleanup Kit (New England Biolabs, T2040L). Purified RNA was quantified using a NanoDrop ND-1000 spectrophotometer (Thermo Fisher Scientific) at 260 nm. Quality was assessed via electrophoresis on a 1.5% agarose gel, ensuring the presence of an intact band. RNA samples were stored at −80°C ultra freezer until further use.

### Reverse transcription PCR analysis of the *phl* gene cluster

Total bacterial RNAs were extracted from the wild-type Pf-5 as described above. The cDNAs were prepared by reverse transcription using SuperScript II reverse transcriptase and random hexamers (Invitrogen, Life Technologies) according to the manufacturer’s instructions. Subsequently, 1 µL of the cDNA reaction mixture was used as the template for PCR amplification. Primers used for PCR analysis of *phl* gene clusters are listed in Table 1. Specifically, transcription of *phlD* was assayed using the primer pair phlD_F and phlD_R. Transcription of *phlA* was assayed using the primer pair phlA_F and phlA_R. Transcription of *phlACB* was assayed using the primer pair phlA_F and phlB_R. Transcription of *phlACBD* was assayed using the primer pair phlA_F and phlD_R. All the PCRs were performed using Q5 Hot Start High-Fidelity 2′ Master Mix (New England Biolabs, Ipswich, MA, USA) under the following conditions: initial denaturation at 98°C for 30 seconds, followed by 30 cycles of denaturation at 98°C for 30 seconds, annealing at 66°C for 30 seconds, and an extension at 72°C for 2 minutes. A final extension step was carried out at 72°C for 5 minutes. PCR products were analyzed by electrophoresis on a 1% agarose gel stained with GelRed nucleic acid stain (Merck, Darmstadt, Germany). Genomic DNA of Pf-5, isolated using the Monarch Genomic DNA Purification Kit (New England Biolabs, Ipswich, MA, USA), was included as a control for the PCR reactions.

### RT-qPCR analysis of *phlD* gene expression

The expression of *phlD* in Pf-5 was analyzed using real-time quantitative PCR. The ∆*gacA* mutant and wild-type Pf-5 were cultured in PDB medium for 24 h. The bacterial total RNA was prepared as described above and used as a template in the RT-qPCR, which was performed using iTaq Universal SYBR Green One-Step Kit (BioRad) on a BioRad CFX96 thermocycler (BioRad). Specifically, the expression of *phlD* was quantified by RT-qPCR using the primer pair phlD_qF/phlD_qR and normalized to the reference gene *zwf* (PFL_4610; glucose-6-phosphate 1-dehydrogenase) using the primer pair zwf_qF/zwf_qR (Table 1). Cycling conditions include reverse transcription reaction at 50°C for 10 minutes, an initial denaturation at 95°C for 1 minute, followed by 40 cycles of 95°C for 10 seconds and 60°C for 30 seconds, with fluorescence acquired at 60°C each cycle. A melt curve was then performed from 65°C to 95°C in increments of 5 seconds per step to verify amplicon specificity. Three biological replicates were used for each treatment. No-template and minus-reverse-transcriptase controls were included and showed no amplification. Cycle threshold values were used for relative quantification of target gene expression, which was calculated in relation to the reference gene using the Pfaffl method (73).

### 5′ RACE and sequencing to identify the transcription start site of the *phlD* gene

Total RNA was extracted from *P. protegens* Pf-5 as described above. cDNA was generated using the NEB Template Switching RT (reverse transcriptase) Enzyme Mix (New England Biolabs) following the manufacturer’s protocol. Briefly, RNA (100 ng) was mixed with a gene-specific reverse primer (phlD_gsp; 1 µM) and dNTPs (1 mM each) and incubated at 70°C for 5 minutes. RT buffer, Template-Switching Oligo (TSO in Table 1; 3.75 µM), and Template Switching RT Enzyme Mix were then added to the annealed RNA. Reactions were incubated at 42°C for 90 minutes and heat-inactivated at 85°C for 5 minutes. TSO-tagged cDNA was then amplified using the TSO-universal primer (TSO-F) and a *phlD* gene-specific reverse primer (phlD_gsp2). PCR cycling conditions were as follows: 98°C for 30 seconds; 5 cycles of 98°C for 10 seconds and 72°C for 30 seconds; 30 cycles of 98°C for 10 seconds, 68°C for 20 seconds, and 72°C for 20–30 seconds; and a final extension at 72°C for 5 minutes. Products were resolved on a 1.5% agarose gel; bands of the expected size (100–500 bp) were excised and purified using the Monarch Gel Extraction Kit (NEB). Purified amplicons were ligated into the linearized pMiniT 2.0 vector using the NEB PCR Cloning Kit (NEB) according to the manufacturer’s instructions. Ligation mixtures were transformed into NEB 10-beta chemically competent *E. coli* cells, recovered in SOC medium (30–60 minutes, 37°C), and plated on LB agar containing ampicillin (100 µg/mL). Colonies were screened by colony PCR, and plasmids from positive clones were prepared using the Monarch Plasmid Miniprep Kit (NEB) and sequenced using kit-supplied vector primers. Chromatograms were trimmed and aligned to the Pf-5 reference genome. A clone was counted as a true 5′ end only if the TSO sequence was present immediately upstream of the genomic sequence with no additional non-templated nucleotides beyond the expected terminal transfer. The transcription start site was defined as the first genomic base 3′ to the TSO-genome junction.

### Bacterial interaction assay

Wild-type Pf-5 and the Δ*gacA* mutant were grown overnight at 28°C in KB liquid. The bacterial cells were washed twice in sterile water, and the cell density was adjusted to an OD_600_ of 1.0. A mycelial plug (5 mm in diameter) of *A. euteiches* was placed at the center of PDA plates. A drop of 3 µL of Pf-5 WT cell suspension (OD_600_ = 1.0) was spotted approximately 2.5 cm from the mycelial plug. The plates were incubated at 28°C for 72 h. The Δ*gacA* mutant was inoculated by spraying the cell suspension (OD_600_ = 1.0) uniformly across the entire hyphal lawn (around 2 mL per plate), and the plates were incubated at 28°C for another 24 h. Following Δ*gacA* colonization, two distinct hyphal regions were developed. Four 6 mm (diameter) agar plugs (zones 1–4) were excised from the culture plate, transferred to sterile tubes, and processed immediately for downstream assays. For hyphal viability assays, each agar plug from zones 1–4 was placed hyphal-side down on fresh PDA plates and incubated at 28°C for 3 days. Regrowth was scored qualitatively: the absence of mycelial outgrowth indicated non-viable hyphae, while robust mycelial expansion indicated viability. Quantification of phloroglucinol was carried out using the cinnamaldehyde-based colorimetric assay (74). Ten agar plugs (~7 mm diameter) were collected and transferred into a flask containing 4 mL of acetone:water (1:1, vol/vol), which was then extracted two times with an equal volume of ethyl acetate. The combined organic phases were vacuum dried, and the dry extracts were resuspended in 100 µL of methanol. A standard curve was prepared using 0.05–10 µg PG solutions in methanol. In a 96-well plate, 100 µL of each standard or sample was mixed with 100 µL of freshly prepared 1% (wt/vol) cinnamaldehyde in ethanol. The plates were incubated at 37°C for 10 minutes to develop the pink-red chromophore, and absorbance was measured at 520 nm using a Spark Multimode Microplate Reader (Tecan). Wells containing water plus reagent alone served as blanks. Phloroglucinol concentrations in samples were determined by interpolation from the standard curve. All measurements were performed in triplicate. The DAPG production was quantified following the same extraction procedure and HPLC analysis described above.

### Statistical analysis

The statistical analysis of the data collected in this study was performed in *R* (version 4.2.3). For two-group comparisons, we used two-sided *t*-tests, applying Welch’s correction by default. For one-factor experiments**,** we used one-way ANOVA. *Post hoc* testing used Tukey’s HSD for all pairwise comparisons.
